# Supplementary material for: The interactome of intact mitochondria by cross-linking mass spectrometry provides evidence for coexisting respiratory supercomplexes
Source: Mol Cell Proteomics. 2017 Dec 8;17(2):216–32. doi: 10.1074/mcp.RA117.000470 (PMC5795388; doi:10.1074/mcp.RA117.000470)
Supplement: Supplemental Data [file supp_RA117.000470_133922_0_supp_23978_fzffwf.zip › spectra_annotation/mito_DR_spectra_annotation/353-1-3-1-5-1.pdf]

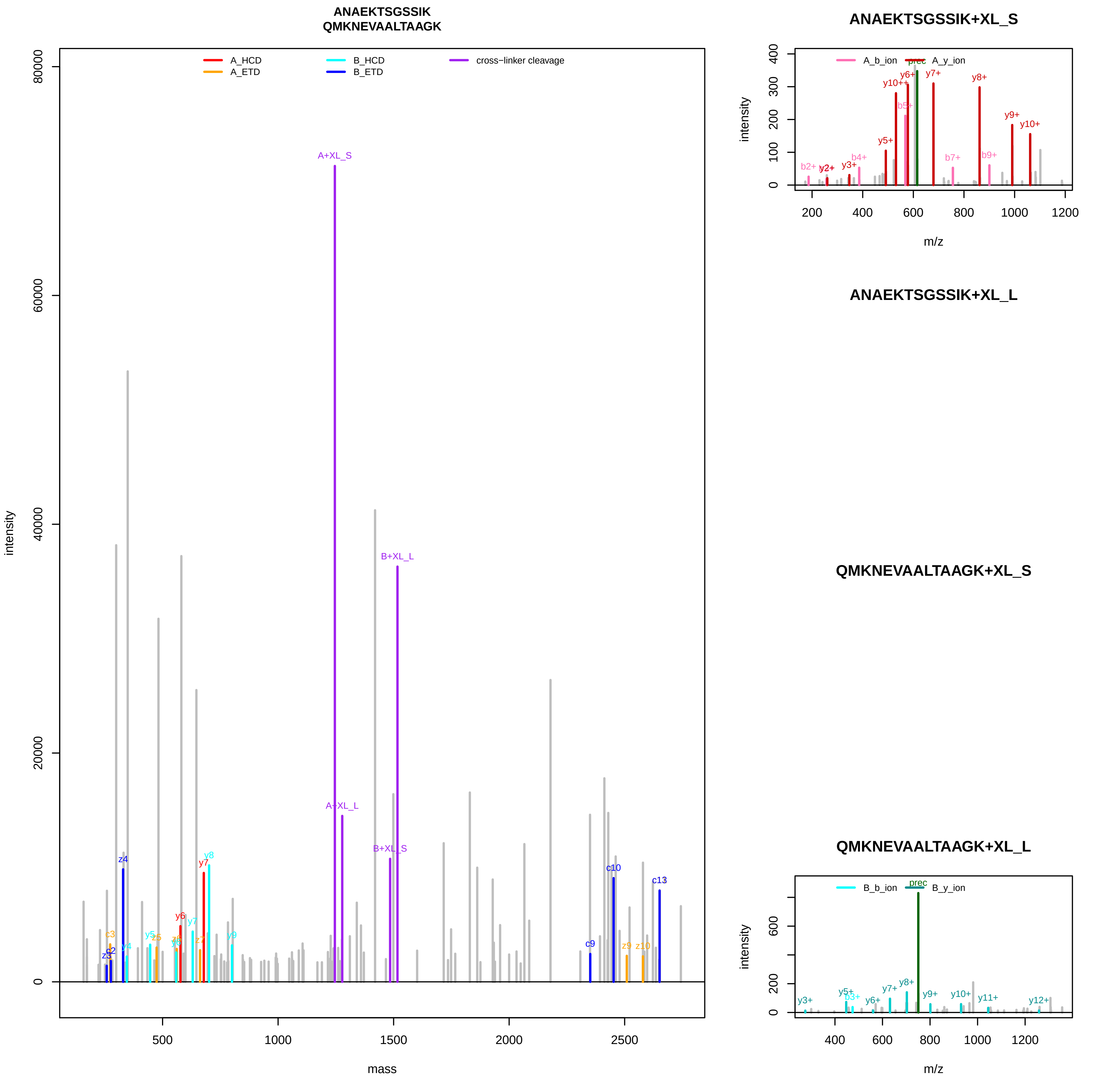
ANAEKTSGSSIK
QMKNEVAALTAAGK
A_HCD
A_ETD
B_HCD
B_ETD
cross−linker cleavage
A+XL_S
B+XL_L
A+XL_L
B+XL_S
intensity
mass
0
20000
40000
60000
80000
500
1000
1500
2000
2500
z4
z3
c2
c3
y4
y5
z5
z6
y6
y7
z7
y8
y9
c9
c10
z9
z10
c13
ANAEKTSGSSIK+XL_S
A_b_ion
A_y_ion
prec
b2+
y2+
y3+
b4+
y5+
y10++
y6+
b5+
y7+
b7+
y8+
b9+
y9+
y10+
m/z
ANAEKTSGSSIK+XL_L
QMKNEVAALTAAGK+XL_S
QMKNEVAALTAAGK+XL_L
B_b_ion
B_y_ion
y3+
y5+
b3+
y6+
y7+
y8+
y9+
y10+
y11+
y12+
